# Spatial Analysis of PAHs in Soils along an Urban–Suburban–Rural Gradient: scale effect, distribution patterns, diffusion and influencing factors

**DOI:** 10.1038/srep37185

**Published:** 2016-11-17

**Authors:** Chi Peng, Meie Wang, Weiping Chen

**Affiliations:** 1 State Key Laboratory of Urban and Regional Ecology, Research Center for Eco-environmental Sciences, Chinese Academy of Sciences, Beijing, 100085, People’s Republic of China

## Abstract

Spatial statistical methods including Cokriging interpolation, Morans I analysis, and geographically weighted regression (GWR) were used for studying the spatial characteristics of polycyclic aromatic hydrocarbon (PAH) accumulation in urban, suburban, and rural soils of Beijing. The concentrations of PAHs decreased spatially as the level of urbanization decreased. Generally, PAHs in soil showed two spatial patterns on the regional scale: (1) regional baseline depositions with a radius of 16.5 km related to the level of urbanization and (2) isolated pockets of soil contaminated with PAHs were found up to around 3.5 km from industrial point sources. In the urban areas, soil PAHs showed high spatial heterogeneity on the block scale, which was probably related to vegetation cover, land use, and physical soil disturbance. The distribution of total PAHs in urban blocks was unrelated to the indicators of the intensity of anthropogenic activity, namely population density, light intensity at night, and road density, but was significantly related to the same indicators in the suburban and rural areas. The moving averages of molecular ratios suggested that PAHs in the suburban and rural soils were a mix of local emissions and diffusion from urban areas.

Polycyclic aromatic hydrocarbons (PAHs) are a group of toxic contaminants widely distributed in the environment^1^. The anthropogenic origins of PAHs includes vehicle exhausts, coal combustion for cooking and heating, wood and straw burning, and high-temperature industries such as melting, coking, and metal processing^2^,^3^. Urban areas with congested traffic, high population densities, and heavy industry are geographic centers of the sources of PAHs^4^. Because soil organic matter (SOM) has a great capacity to adsorb PAHs in the environment^5^, they are prone to deposition on soil surfaces and can persist in soil for decades^6^,^7^. Knowledge of the distribution patterns of PAHs and of their composition in soil is useful in identifying the sources of PAHs, in risk assessment, and in studying the pollution history of a city^8^,^9^.

The spatial distribution of PAHs has been shown to vary with scale^10^,^11^. On a continental scale, the distribution of PAHs in soil reflects regional differences in the degree of economic development and population density^12^,^13^,^14^. For instance, soil PAH concentrations in eastern China are higher than those in western China because of unbalanced development^15^. On a regional scale, the distribution of PAHs is associated primarily with the expansion of urbanization^16^. The concentrations of soil PAHs are reported to decrease along the urban–suburban–rural gradient^17^,^18^,^19^. Point sources such as industrial plants and mines, which can contaminate isolated spots, are apparent only when contamination is mapped on a regional scale; on continental-scale maps, such point sources usually remain undetected^20^. On a block scale or even finer scales, the complex patterns of the distribution of PAHs, influenced by multiple factors, become evident. Concentrations of soil PAHs change with land use, being always higher around industrial areas and locations close to main roads and lower in agricultural soils and vacant lots^21^,^22^. Vegetation canopy structure, height of buildings, and the structure of urban areas affect the flux of atmospheric depositions and thus also affect PAH concentrations in soils^23^,^24^. Soil physical disturbance during changes in land use and maintenance of green spaces may change concentrations of soil PAHs dramatically^4^,^25^. Soils sampled from windward and leeward sides of a house showed large differences in the concentrations of pollutants^26^, and the distance from road networks had a significant impact on concentrations of PAHs in soil^27^. Therefore, such scale-effects should be taken into account in studying the spatial patterns of the distribution of PAHs and the factors that influence the accumulation of PAHs.

Spatial statistical methods developed for mineral exploration have been deployed for studying spatial distribution of soil contaminants in urban and peri-urban areas^28^. Different from classical statistics, spatial statistical methods focus on the spatial characteristics of variables, namely spatial autocorrelation, spatial heterogeneity, spatial clustering, and spatial relationships. Spatial interpolation methods, such as Inverse Distance Weighted (IDW) and Kriging family, which can model the spatial autocorrelation of contaminants in soils, are capable of predicting pollution levels at a given location without physical measurements^29^. Such interpolation methods are widely used for mapping probable concentrations of soil contaminants on continuous surfaces and to reveal spatial processes^11^,^24^,^30^,^31^. If the prediction errors are substantial because of low sampling density or because of the complex environment of urban and suburban areas^32^, Cokriging interpolation could be used instead, which makes predictions more accurate by taking into consideration the correlations between the target contaminant and other factors such as soil properties^33^.

Other spatial statistical methods, such as Getis-ord Gi* analysis and Morans I analysis, study spatial clusters and spatial heterogeneity of soil contaminants^34^. Earlier studies have adopted Morans I analysis to identify pollution hotspots and spatial outliers based on results of soil surveys^35^,^36^. The geographically weighted regression (GWR) method, a spatial form of linear regression, analyzes spatial relationships between variables^37^. Better than the classical regression method, GWR can be used for exploring spatially varied relationship between soil contaminants and the factors that influence the extent of contamination, such as population distribution, traffic distribution, urban development levels, soil properties, and land-use types. Extra information can be obtained from ‘spatializing’ the classical statistical results, that is by mapping the moving average, median, and standard deviation values for the concentration of contaminants in neighborhood soils^38^. In other words, spatial statistical methods offer a spatial perspective to analyse the sources, distribution, and causes of soil contamination. However, spatial statistics have rarely been applied systematically for studying soil pollution because of the lack of adequate sampling density or the lack of expertise in spatial statistical methods.

China’s extensive economic growth has led to a substantial increase in emissions of PAHs, and rapid urbanization has led to frequent changes in land use and fragmentation of the landscape. For better urban planning and pollution control, it is essential to describe the impacts of urbanization on the distribution of soil PAHs systematically. It was against this background that we built a large data set of PAH concentrations in urban, suburban, and rural soils of Beijing and also collected spatial indicators of the intensity of anthropogenic activity as population density, road density and light intensity at night in the study areas. Several GIS-based spatial statistical methods were integrated to analyse the sources, movement, influencing factors and distribution patterns of PAHs in urban, suburban, and rural soils. Briefly, these spatial statistical methods include (1) Cokriging analysis to map the spatial distribution of soil PAHs, (2) Anselin local Moran’s I analysis to highlight the spatial hotspots and outliers of soil PAH concentrations, (3) Global Moran’s I analysis to reveal the patterns of spatial autocorrelation for soil PAHs, (4) GWR to study the spatial correlations between soil PAH concentrations and intensity of anthropogenic activities, and (5) the moving statistics to visualize the spatial variance and general trend of soil PAHs accumulation along the urban-suburban-rural gradient.

## Material and Methods

### Study area and data collection

Beijing is one of the largest cities in China and has grown rapidly over the past 40 years. The urban sprawl of Beijing started at the Forbidden City has led to six concentric ring roads. Generally, the areas (districts) inside the 5th ring road are considered to be the urban areas of Beijing; those between the 5th and the 6th ring road make up the suburban areas of Beijing; and areas outside the 6th ring road form the rural areas of Beijing. We collected two data sets of PAH concentrations in surface soils, one comprising 233 soil samples (collected in September 2008) representing the urban areas and the other comprising 243 soil samples (collected in September 2010) representing the suburban and rural areas (the rural areas were part of Tongzhou district outside the 6th ring road) (Fig. S1 and Fig. S2 in the supplementary information). Each sample was the equal composite of five subsamples obtained from an appropriate 10 m × 10 m area close to the designated sampling site. Surface soils (0–10 cm) were taken by a stainless steel hand auger and were transported to the laboratory in plain paper bags. Generally, concentrations of PAHs in soil were measured through a three-step process involving extraction, purification, and quantification. The soil PAHs and soil properties such as soil organic carbon (SOC) content and pH were measured following the procedures described elsewhere^22^,^39^.

To quantify the spatial distribution of the intensity of anthropogenic activity, we mapped population density on the scale of a block, taking data from the census conducted in 2010^40^. The light intensity at night can be considered a proxy for the intensity of anthropogenic activity, and a high-resolution map of night light intensity was extracted from remote-sensing data from the visible infrared imaging radiometer suite (VIIRS) day/night band (DNB) (http://ngdc.noaa.gov/eog/index.html). The road density of Beijing represented as the total road length (kilometers) divided by the searching area (square kilometers) was calculated based on remote-sensing data using a scanning radius of 500 m.

### Spatial statistical analyses

Statistical analyses, including the Kolmogorov–Smirnov test and the Pearson correlation analysis, were conducted using SPSS ver. 18.0. The PAH data and spatial data were log-transformed before the statistical analyses. One outlier – a wood preservation plant that recorded total PAH concentrations of 78 476 ng/g – was excluded from the statistical analyses and is discussed elsewhere^22^.

The concentrations of PAHs were mapped, as a covariate of SOC, using the Cokriging interpolation method. The Stable function was selected as the semivariogram model and was set to anisotropy mode. Settings of the Cokriging method were adjusted in order to provide the lowest prediction errors in the cross-validation process which repeatedly removes one data point at a time than predicts the omitted data value based on the rest of the data. The results of cross validation and the plots of semivariogram and covariance were shown in the Table S1 and Fig. S3 (supplementary information). Global Moran’s I analysis with incremental distance and the Anselin local Morans I analysis were conducted to analyse the spatial patterns, clusters, and outliers of soil PAHs. The spatially varying relationships between concentrations of PAHs in soil and the indicators of the intensity of anthropogenic activity were analysed by the GWR method based on a fixed neighbor number of 30. The spatial distribution of neighbourhood statistics was mapped based on voronoi polygons in which each assigned value was the local standard deviations calculated from the polygon and its neighbors. Geostatistical analyses and spatial statistical analyses were carried out using ArcGIS ver. 10.1.

**Figure 1:**
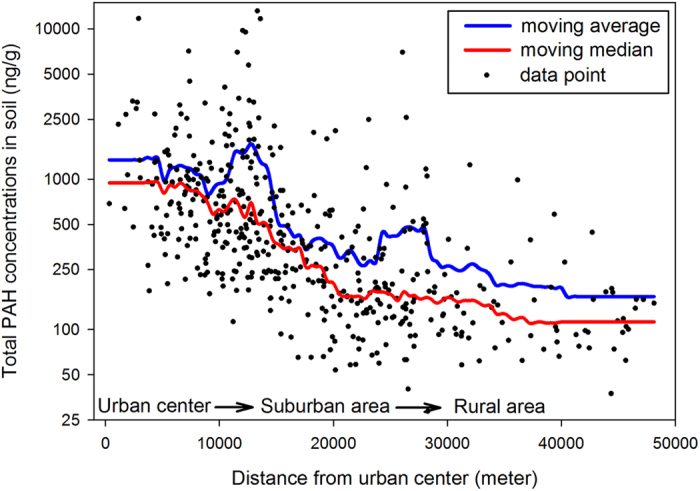
The mean and median concentrations of PAHs along the urban-suburban-rural gradient of Beijing.

The moving statistical values of the concentrations of PAHs in soil along the urban–suburban–rural gradient were plotted using Sigmaplot ver. 12.0. The distance from urban center in Fig. 1 was calculated as the distance from sampling site to the Forbidden City (Fig. S1). The moving statistical curves were composed of 100 uniformly spaced intervals along the X-axes and the value in each interval was sampled by the nearest 10% neighbors (47 data points).

## Results and Discussion

### Regional patterns of PAH distribution

The urban sprawl of Beijing starts from the city center, namely the Forbidden City, and expands outwards to encompass six ring roads (Fig. S1). Total concentrations of 16 PAHs – specified by the US Environmental Protection Agency – in soil showed a clearly decreasing pattern along the urban–suburban–rural gradient (Fig. 1). The mean and median values of total PAH concentrations were 1228 ng/g and 688 ng/g in urban soils, 322 ng/g and 189 ng/g in suburban soils, and 219 ng/g and 145 ng/g in rural soils, respectively. The moving average curve of total PAH concentration showed two peaks at 13000 and 26000 m distances because of the industrial factories around the 5^th^ ring road and the 6^th^ ring road. The total PAH concentrations showed a log normal distribution in the study area (passed the K–S test) and their mean values were higher than their median values at any distance from the urban center to the rural area (Fig. 1). The difference between the mean and the median values suggests that the distribution of soil PAH concentrations was skewed due to anthropogenic activity in urban, suburban, and rural areas. The concentrations of individual PAH congeners were closely related to total PAH concentrations (Table S2), which means that they had similar spatial distributions.

**Figure 2:**
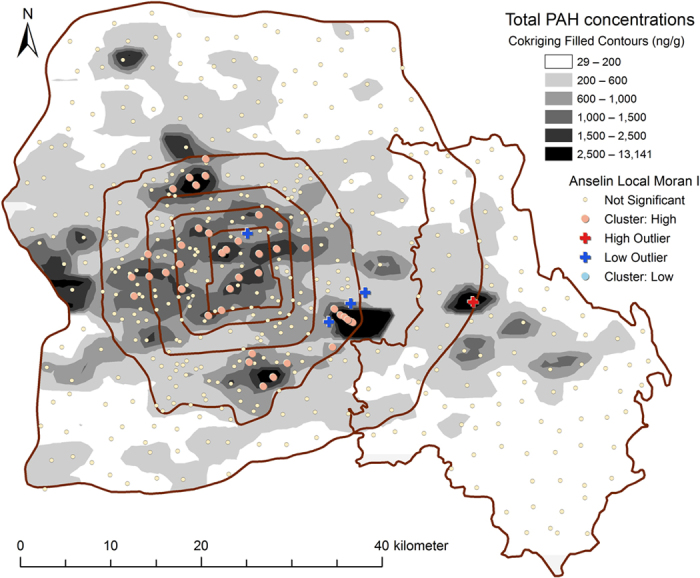
Sptail distribution of PAHs in the urban, suburban and rural soils of Beijing based on the Cokriging and the Anselin Local Moran I analysis (created by ArcGIS 10.1, http://www.esri.com/software/arcgis/arcgis-for-desktop).

As shown in Fig. 2, the spatial distribution of PAH concentrations in soil is a combination of two patterns, namely (1) regional differences between the urban, suburban, and rural areas and (2) isolated high-pollution hotspots. The total PAH concentrations were higher than 600 ng/g in most of the urban areas and lower than 200 ng/g in most of the rural areas (Fig. 2). Polycyclic aromatic hydrocarbons emitted from non-point sources and mobile sources mix in the air and are eventually deposited on the ground to form regional baseline deposits^4^. The differences in soil PAH concentrations between the urban, suburban, and rural areas can be explained by changes in baseline deposits between these areas and are most probably related to regional levels of urban development. The sampling sites of rural area were located in the downwind area of Beijing (southeast Beijing) which may receive PAH diffusions from the urban areas (Fig. S2). However, further statistical analysis is needed due to lack of direct evidence of PAHs transport from urban to rural areas only from the map of PAH concentrations in the soils.

Potential point sources of PAHs are scattered around the city of Beijing and include an iron and steel corporation, coking and carbide factories, three power plants, an automobile plant, a chemical plant, a military airfield, and a civilian airport (Fig. S2). Isolated hotspots of high PAH concentrations were found near these point sources (Fig. 2), except the two power plants inside the 5th Ring road and the civilian airport to the north-west. Once released from such major stationary sources, PAHs end up in surrounding soils, mainly through atmospheric deposition. These processes would significantly elevate PAH concentrations in the neighborhood soil. The number of sampling sites that are under the influence of such point sources depends on sampling density. In the present study, the sampling site closest to the international civilian airport was at least 3 km away from the runway and therefore recorded marginally higher PAH concentrations than the surrounding sites. Although the sampling density for the urban areas was higher, soil PAH concentrations near the two power plants that were part of the urban areas were not significantly higher (Fig. S2). These power plants are expected to have the strictest emission control measures because they are within the city limits of Beijing, and PAHs released from the two power plants were no longer easily distinguishable from the baseline deposits for the urban area. The other point sources, because of their high energy consumption, account for the considerably higher PAH concentrations in the surroundings sites.

### Effect of scale on the distribution of PAHs

Anselin Local Moran’s I analysis was used for identifying spatial outliers and spatial clusters of sampling sites with high PAH concentrations. Such spatial clusters of high polluted sites were marked as hotspots and were concentrated in the old urban areas (Fig. 2), including the city center, the educational district, and the south-west industrial district. The old urban areas have high levels of PAHs from non-point sources including densely populated pockets, heavy traffic, and traffic congestion and also because of their long history of development. The heavy industrial plants in the south-west industrial district were recently moved out of the city; however, the levels of PAHs in the soil remain high.

Anselin Local Moran’s I analysis identified five spatial outliers for soil PAHs (Fig. 2), including one high-low site (a high polluted site surrounded by several low polluted sites) and four low-high sites (a low polluted site surrounded by several high polluted sites). The high-low site was a private backyard used for growing vegetables in a small village near a chemical plant (Fig. S2), and the high PAH concentrations may have been due to application of sludge or wastewater. The low-high sites in urban areas have been less discussed in earlier studies. Peng *et al*.^4^ found that physical disturbances to soil that accompany changes in land use can alter the PAH concentrations in surface soil dramatically, either by removing the surface layer or by covering it with fresh soil. Although exposed to high levels of baseline deposits, the four low-high sites had relatively low concentrations of PAHs, probably due to lawn renovation or change in land use type in recent years.

**Figure 3:**
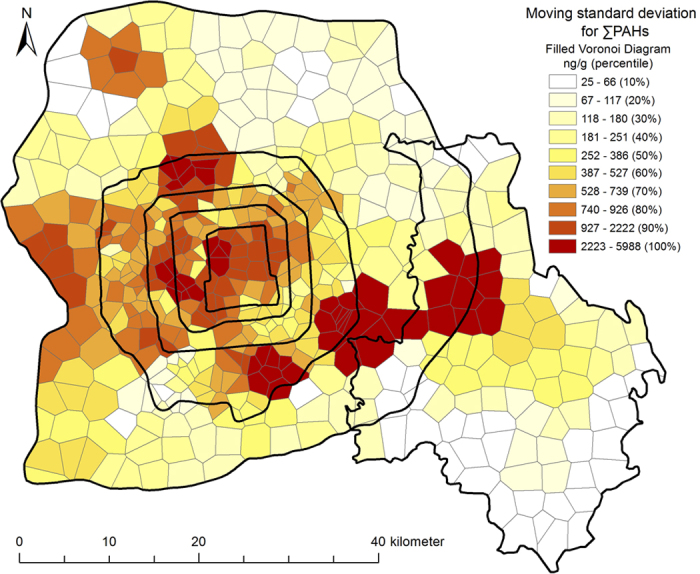
Spatial distribution of standard deviation of the soil PAHs along the urban-suburban-rural gradient of Beijing (created by ArcGIS 10.1, http://www.esri.com/software/arcgis/arcgis-for-desktop).

Spatial distribution of standard deviations of soil PAHs is shown in Fig. 3. High standard deviations appear in the urban areas and in the neighborhood of point sources. Large differences in soil PAH concentrations are expected between areas within and outside the sphere of influence of point sources. The high standard deviations of PAH concentrations in urban areas resulted from multiple causes, such as changes in vegetation cover and land use, proximity to roads with heavy traffic, and physical disturbances to soil^4^,^23^. Although these may have been the causes of the high variability in soil PAHs in urban areas, the overall distribution of PAHs was consistent with the regional baseline deposits (Fig. 2). The results suggest a scale effect on the distribution of soil PAHs along the urban–suburban–rural gradient: on a regional scale, the distribution was predominantly affected by the level of urbanization and proximity to point sources, whereas on the scale of a block, the predominant factors were the underlying surface, vegetation, land use, physical disturbance, and microclimate.

### Spatial correlation with the intensity of anthropogenic activity

Concentrations of PAHs in the environment result mainly from anthropogenic activity. We adopted three quantitative indicators to describe the spatial distribution of the intensity of such activity along the urban–suburban–rural gradient of Beijing, namely population density, light intensity at night, and road density—all of which were significantly correlated to concentrations of PAHs in soil (*p* < 0.01, Table S3). The main source of PAHs was combustion, whether for heating, cooking, or motorized transport. Higher density of population and of traffic therefore leads to higher PAH deposition in the neighborhood. However, these correlations may vary spatially and become less significant if the soil PAHs are predominantly regional baseline deposits or deposits from point sources.

**Figure 4:**
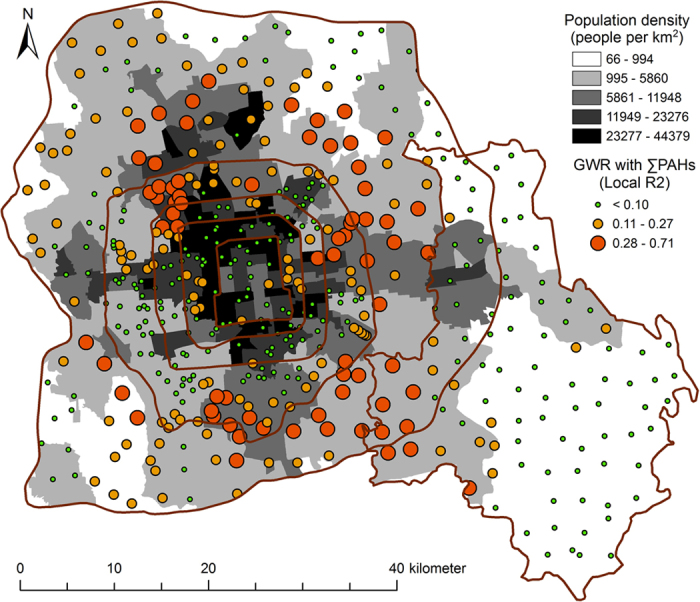
Local *R*^*2*^ of GWR analysis between total PAH concentrations and population density in the urban, suburban, rural gradient of Beijing based on fixed 30 neighbors (created by ArcGIS 10.1, http://www.esri.com/software/arcgis/arcgis-for-desktop).

To ascertain whether these correlations spatially vary, we used the GWR method of analysis in which the local *R*^*2*^ values indicated how well the neighborhood variables can predict the concentrations of PAHs in soil. Mapping the local *R*^*2*^ values (e.g. the regression between concentrations of total PAHs in soil and population density in the corresponding areas of Beijing) can help us to further understand how anthropogenic activity can affect the distribution of PAHs in different regions. The local values of *R*^*2*^ between PAH concentrations and population density are shown in Fig. 4. Mostly, the correlations were high in suburban areas and low at the city center and in rural areas. The high local *R*^*2*^ values in the suburban areas suggest that the level of urbanization is the dominant factor that determines PAH concentrations in those areas. Increases in population in the newly developed areas would elevate the concentrations rapidly in the neighborhood owing to increased traffic, heating, and cooking. The spatial resolution of population density in the rural areas was too low to show any definitive effect of population density on the concentrations of PAHs.

**Figure 5:**
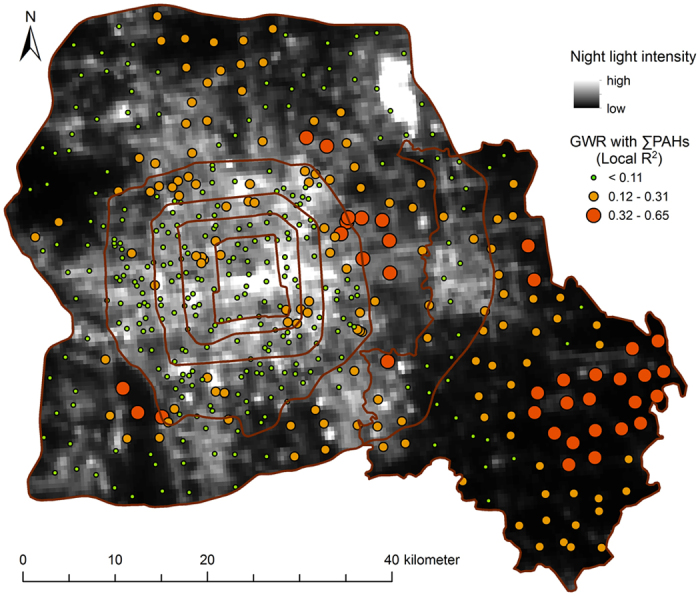
Local *R*^*2*^ of GWR analysis between total soil PAH concentrations and night light intensity in the urban, suburban, rural gradient of Beijing based on fixed 30 neighbors (created by ArcGIS 10.1, http://www.esri.com/software/arcgis/arcgis-for-desktop).

**Figure 6:**
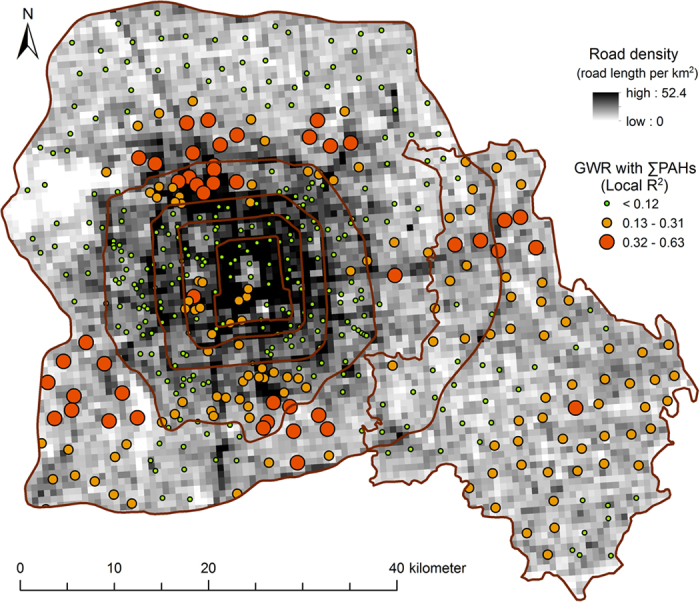
Local *R*^*2*^ of GWR analysis between total soil PAH concentrations and road density in the urban, suburban, rural gradient of Beijing based on fixed 30 neighbors (created by ArcGIS 10.1, http://www.esri.com/software/arcgis/arcgis-for-desktop).

The correlation between PAH concentrations and light intensity at night was high mostly in suburban areas and rural areas, and low in urban areas and in areas close to the point sources (Fig. 5). The data on light intensity extracted from satellite images had a higher resolution than the data on population density and were better indicators of the intensity of anthropogenic activity. In the suburban and rural areas, the sources of illumination at night were mostly residential areas, rural settlements, and transportation systems. These results suggest that daily domestic routines and traffic were the most important factors affecting the distribution of PAHs in suburban and rural areas. Similarly, values of the correlation between concentrations of PAHs in soil and road density were also found to be high in the suburban and rural areas of Beijing (Fig. 6), emphasizing the importance of traffic in the accumulation of PAHs in these areas.

In urban areas, the correlation between concentrations of PAHs and any of the indicators of the intensity of anthropogenic activity – population density, light intensity at night, and road density – was low (Fig. 4, 5, 6). The intensity of anthropogenic activity represented by the indicators was relatively evenly distributed in the urban areas, probably because PAHs released from urban areas form citywide baseline deposits^4^. The spatial heterogeneity of land use and vegetation cover as well as physical disturbance to soil may mask the correlations between soil PAHs and the indicators of the intensity of anthropogenic activity^23^: significant correlations between concentrations of PAHs in soil and population density can be found in urban areas only when the land use is uniform, such as residential areas^4^,^23^. Therefore, we should pay more attention to influences that operate on smaller scales in studying pollution in urban areas.

### Sources and movement of PAHs

**Figure 7:**
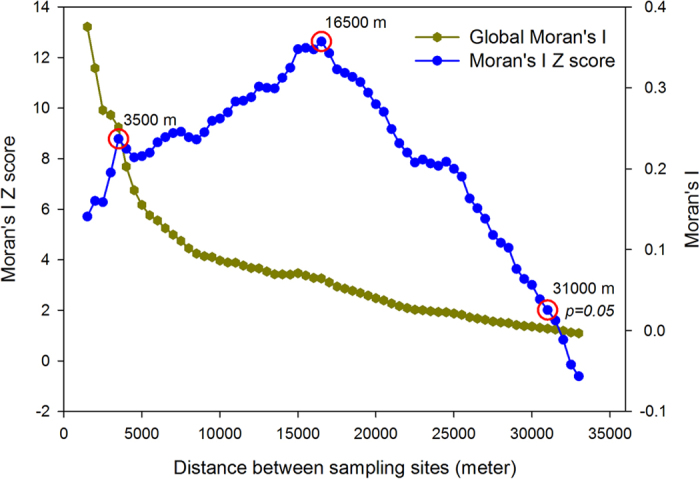
Global Moran’s I values and z-scores of soil PAH concentrations along the urban-suburban-rural gradient of Beijing.

Global Moran’s I analysis with incremental distance was undertaken to identify the general spatial autocorrelation pattern of concentrations of PAHs in soil along the urban–suburban–rural gradient in Beijing. The Global Moran’s I values decreased with distance between sampling sites whereas the z-scores showed two peaks, one at 3.5 km and another at 16.5 km (Fig. 7). The Global Moran’s I values indicate that concentrations of PAHs in soil were positively correlated to neighborhood measurements and the peak z-scores suggest that the distances at which spatial processes promote clustering are the most significant. The two peaks indicated two spatial processes most likely to cause two distinct spatial patterns; in other words, the peak at 3.5 km suggests the sphere of influence of point sources and that at 16.5 km suggests the effective reach of regional baseline deposits.

The molecular ratios of PAH congeners vary with emission sources and are widely used for source apportionment^41^,^42^. Table 1 shows that the mean ratios of CombPAHs/total PAHs, FLT/(FLT+PYR), IND/(IND+BghiP), BaA/(BaA+CHR), and BaP/BghiP in the urban, suburban, and rural soils of Beijing were 0.77, 0.56, 0.50, 0.38, and 0.86, respectively. All these ratios were higher than their corresponding diagnostic values for combustion sources, which are as follows: 0.7 for CombPAHs/Total PAHs, 0.4 for FLT/(FLT+PYR), 0.2 for IND/(IND+BghiP), 0.35 for BaA/(BaA+CHR), and 0.6 for BaP/BghiP^5^. These ratios suggest that the predominant source of PAHs was combustion—of coal for heating and cooking, of biomass in rural areas, and of liquid fossil fuels for motorized transport.

**Figure 8:**
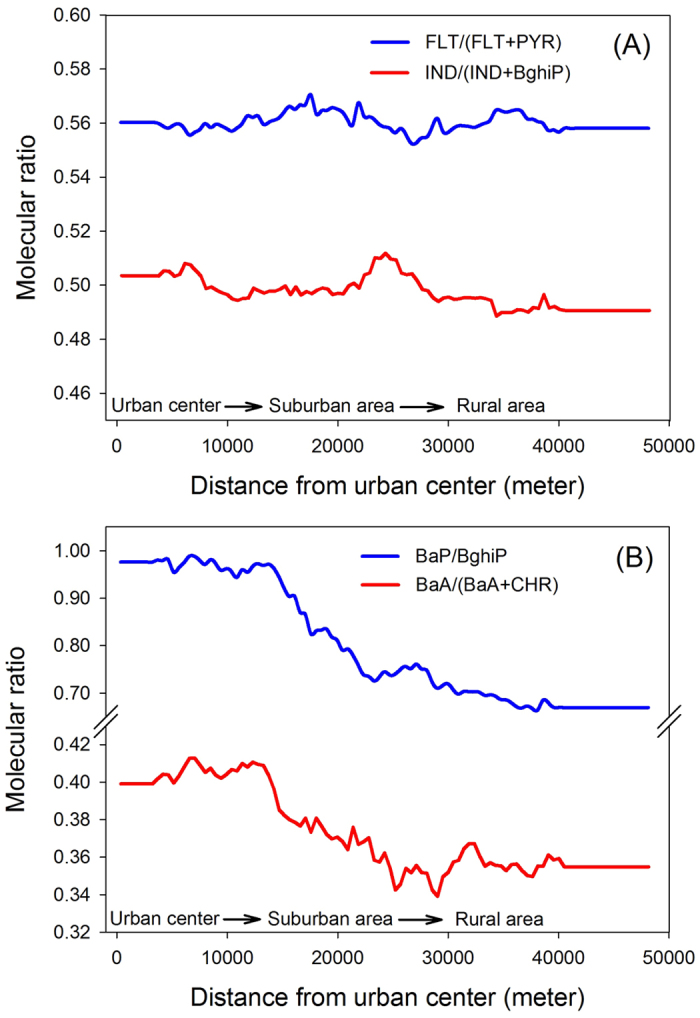
Moving averages of PAH molecular ratios along the urban-suburban-rural gradient of Beijing.

The ratios of FLT/(FLT+PYR) and of IND/(IND+BghiP), depending on whether they are higher or lower than 0.5, can further distinguish between liquid fossil fuel combustion and biomass combustion^3^. These two molecular ratios rarely changed along the urban–suburban–rural gradient of Beijing (Fig. 8A) and were unrelated to any of the three indicators of the intensity of anthropogenic activity (Table 1), which suggests that PAHs in the soils had common sources despite the differences in land use at the sampling sites. The land-use type and distance from source can affect the concentrations of PAHs in soil but can hardly change the ratios of FLT/(FLT+PYR) and IND/(IND+BghiP) because the two ratios remain stable during transport through air.

The ratios of BaA/(BaA+CHR) and BaP/BghiP showed a different distribution pattern: the ratios took the form of horizontal lines in urban areas, dipped sharply in suburban areas, and remained relatively low in rural areas (Fig. 8B). The ratios were also significantly related to the indicators of the intensity of anthropogenic activity (Table 1). BaA and BaP photo-degrade faster than their isomers; therefore, BaA/(BaA+CHR) and BaP/BghiP ratios tends to change as a result of atmospheric photoreactions^3^,^41^. The unchanged ratios of FLT/(FLT+PYR) and IND/(IND+BghiP) suggest that the sources of PAHs were common to the three areas, namely urban, suburban, and rural, whereas the decreasing values of the BaA/(BaA+CHR) and BaP/BghiP ratios indicate airborne transport of PAHs from urban areas to rural areas. Similarly, significant positive correlations were found between CombPAHs/Total PAHs and population density, light intensity at night, and road density (Table 1). These correlations prove that PAHs in suburban and rural soils were a mix of local emissions and diffusions from urban areas. In additional, the ratios of BaA/(BaA+CHR) and BaP/BghiP in the hotspot sites (the high cluster and high outlier sites in Fig. 2) were higher than their mean values of total sites, while the FLT/(FLT+PYR) and IND/(IND+BghiP) ratios had little change (Table 1). The increased BaA/(BaA+CHR) and BaP/BghiP ratios suggested that the hotspot sites were receiving PAHs deposits mainly from local emission sources.

## Conclusions

In summary, the results suggest that the distribution of PAHs on a regional scale is affected by the level of urbanization and industrial point sources (within a distance of 16.5 km and 3.5 km, respectively). The distribution of PAHs in urban areas was unrelated to population density, light intensity at night, and road density. The high spatial heterogeneity of PAHs in soil on the scale of a block was mainly due to spatial and temporal changes in vegetation cover and land use. In the suburban and rural areas, PAHs in soil are a mix of local emissions from combustion and diffusion from urban areas and were significantly correlated to the indicators of the intensity of anthropogenic activity.

## Additional Information

**How to cite this article**: Peng, C. *et al*. Spatial Analysis of PAHs in Soils along an Urban–Suburban–Rural Gradient: scale effect, distribution patterns, diffusion and influencing factors. *Sci. Rep.* **6**, 37185; doi: 10.1038/srep37185 (2016).

**Publisher’s note:** Springer Nature remains neutral with regard to jurisdictional claims in published maps and institutional affiliations.

## Supplementary Material

Supplementary Information

## Figures and Tables

**Table 1:** PAH molecular ratios and their correlations with the indicators of human activity intensity.

	Total sites (Mean ± SD)	Hotspot sites^a^ (Mean ± SD)	Population density	Night light intensity	Road density
CombPAHs^b^/Total PAHs	0.77 ± 0.07	0.83 ± 0.05	0.300**	0.273**	0.356**
FLT/(FLT+PYR)	0.56 ± 0.02	0.55 ± 0.02	0.011	−0.016	−0.07
IND/(IND+BghiP)	0.50 ± 0.03	0.51 ± 0.02	0.081	0.056	−0.002
BaA/(BaA+CHR)	0.38 ± 0.07	0.44 ± 0.03	0.323**	0.311**	0.379**
BaP/BghiP	0.86 ± 0.19	1.07 ± 0.15	0.575**	0.512**	0.501**

^**^Correlation is significant at the 0.01 level (2-tailed).

^a^Hotspot sites denote the high cluster and high outlier sites according to the Anselin local Moran I analysis.

^b^CombPAHs: combustion PAH, sum of FLT, PYR, BaA, CHR, BkF, BbF, BaP, IND and BghiP.
