# Supplementary material for: Spatial Analysis of PAHs in Soils along an Urban–Suburban–Rural Gradient: scale effect, distribution patterns, diffusion and influencing factors
Source: Sci Rep. 2016 Nov 17;6:37185. doi: 10.1038/srep37185 (PMC5112551; doi:10.1038/srep37185)
Supplement: Supplementary Information [file srep37185-s1.pdf]

Supporting information

Spatial Analysis of PAHs in Soils along an Urban–Suburban–Rural Gradient: scale effect, distribution patterns, diffusion and influencing factors

Chi Peng, Meie Wang, Weiping Chen *

State Key Laboratory of Urban and Regional Ecology, Research Center for Eco-environmental Sciences, Chinese Academy of Sciences, Beijing, 100085People's Republic of China

* corresponding author

Tel: +86-010-62843981

E-mail address: wpchen@rcees.ac.cn (*W.P. Chen*)

List of contents

Table S1 Results of cross validation in the Cokriging interpolation analysis

Table S2 Correlations between individual PAH congeners and the total PAH concentrations

Table S3 Correlations between soil PAH concentrations and the indicators of human activity intensity

Fig.S1 The distribution of sampling sites in the urban, suburban, and rural areas of Beijing (created by ArcGIS 10.1, http://www.esri.com/software/arcgis/arcgis-for-desktop)

Fig.S2 The distribution of land uses and point sources in the urban, suburban, and rural areas of Beijing (created by ArcGIS 10.1, http://www.esri.com/software/arcgis/arcgis-for-desktop)

Fig.S3 Semivariogram and covariance plots with different search directions in the Cokriging interpolation analysis

Table S1 Results of cross validation in the Cokriging interpolation analysis

Prediction Errors	
Mean error	-28.148
Root mean square error	1080.114
Mean Standardized Error	-0.134
Median Standardized Error	0.109
Root mean square standardized error	1.007

Table S2 Correlations between individual PAH congeners and the total PAH concentrations

	∑PAHs	LMW PAHs	HMW PAHs
NAP	.560**	.680**	.525**
ACPY	.913**	.896**	.907**
ACP	.884**	.904**	.869**
FL	.892**	.935**	.872**
PHE	.961**	.981**	.947**
ANT	.976**	.960**	.970**
FLT	.988**	.949**	.987**
PYR	.990**	.947**	.989**
BaA	.990**	.930**	.993**
CHR	.982**	.932**	.983**
BbF	.982**	.913**	.987**
BkF	.941**	.882**	.945**
BaP	.986**	.915**	.992**
IND	.983**	.913**	.988**
DBA	.982**	.914**	.986**
BghiP	.982**	.913**	.987**

**. Correlation is significant at the 0.01 level (2-tailed).

Table S3 Correlations between soil PAH concentrations and the indicators of human activity intensity

	Total PAHs	Distance from urban center	Road density	Night light intensity	population density	population growth rate
Total PAHs	1	-0.592**	.569**	.536**	.608**	.133**
LMW PAHs	.959**	-0.586**	.554**	.545**	.621**	.162**
HMW PAHs	.997**	-0.590**	.569**	.531**	.600**	.125**
Carc-PAHs	.995**	-0.575**	.557**	.518**	.585**	.120**
Comb-PAHs	.997**	0.591**	.569**	.531**	.601**	.125**

**. Correlation is significant at the 0.01 level (2-tailed).

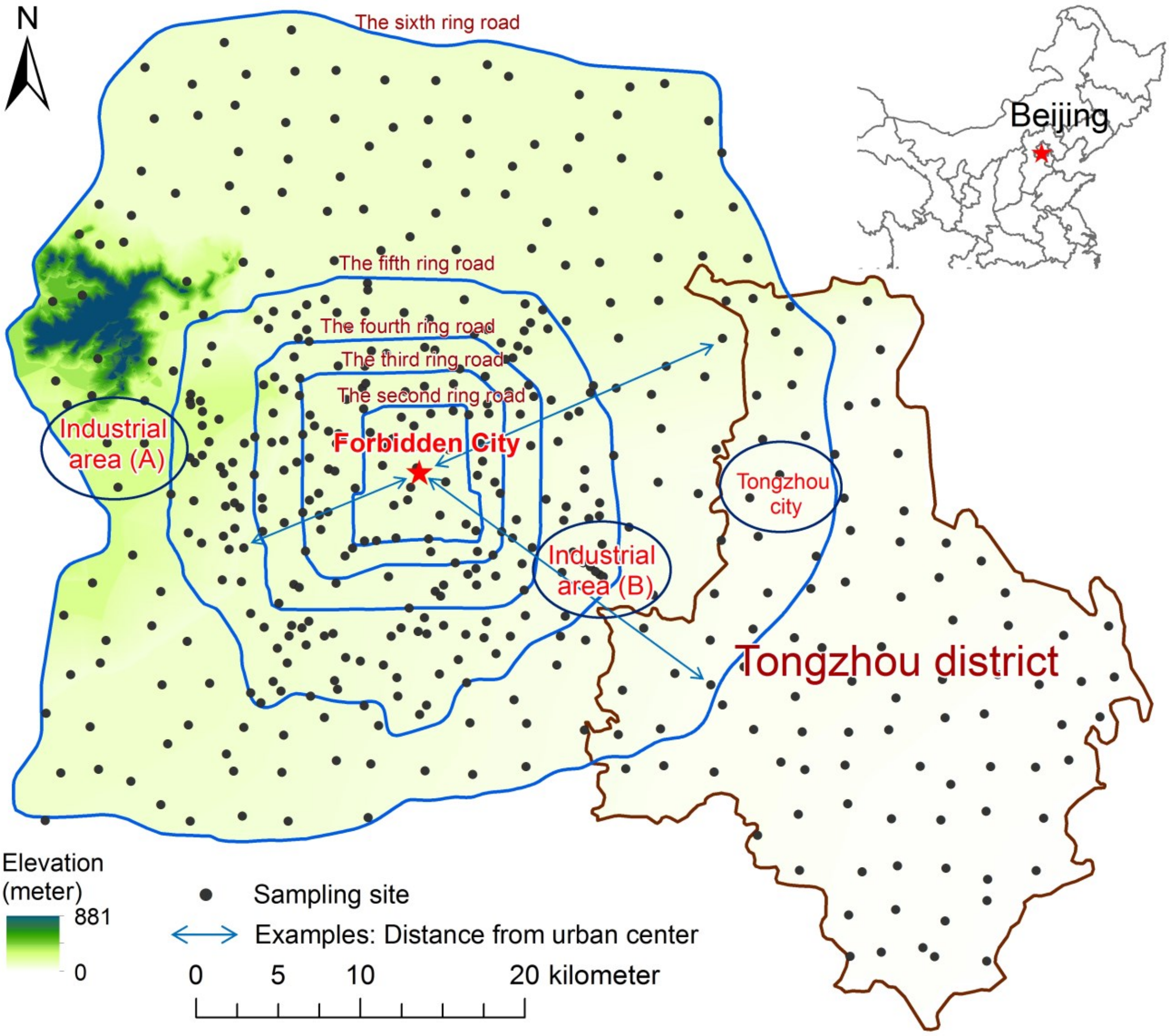

Fig.S1 The distribution of sampling sites in the urban, suburban, and rural areas of Beijing (created by ArcGIS 10.1, http://www.esri.com/software/arcgis/arcgis-for-desktop)

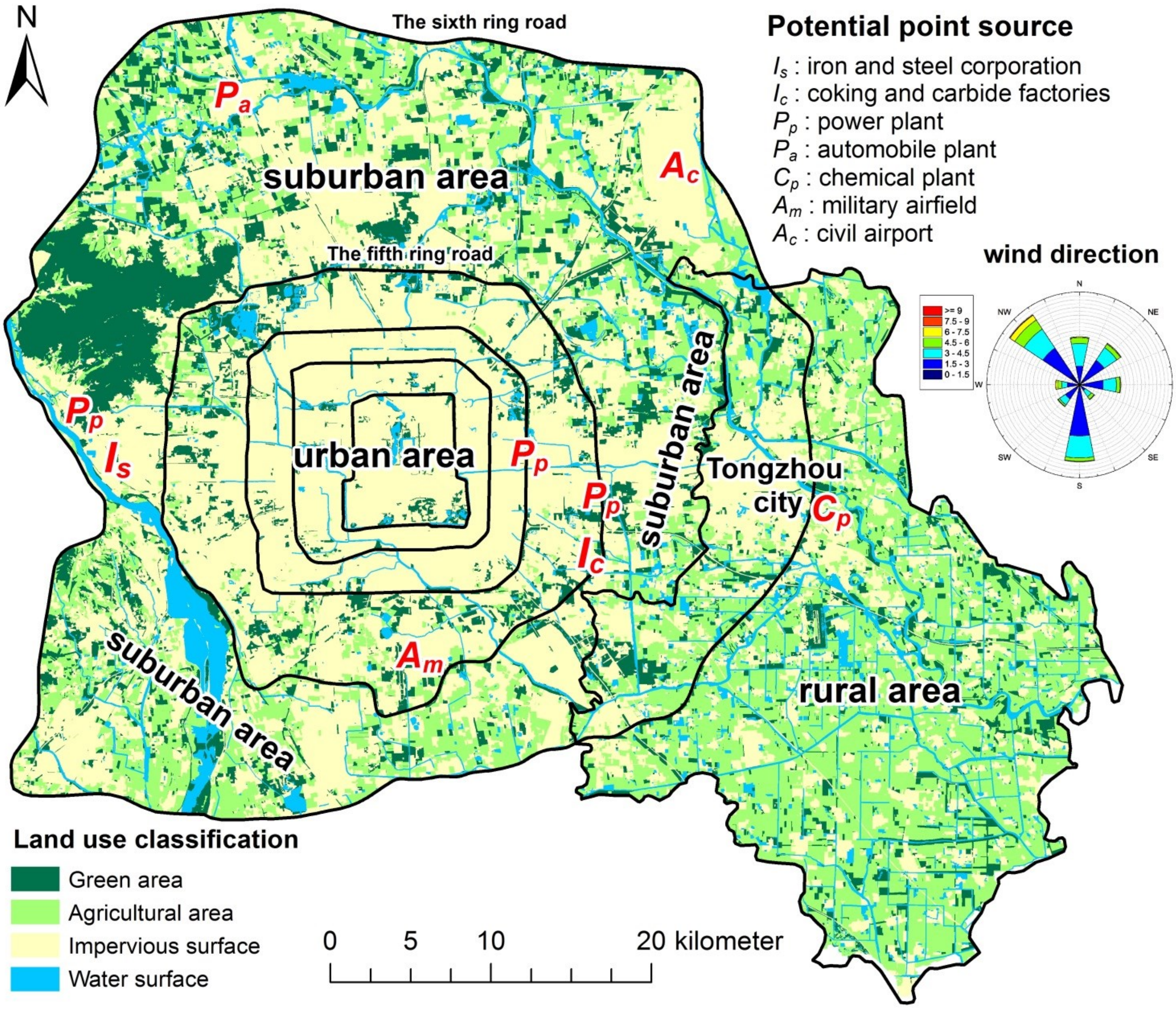

Fig.S2 The distribution of land uses and point sources in the urban, suburban, and rural areas of Beijing (created by ArcGIS 10.1, http://www.esri.com/software/arcgis/arcgis-for-desktop)

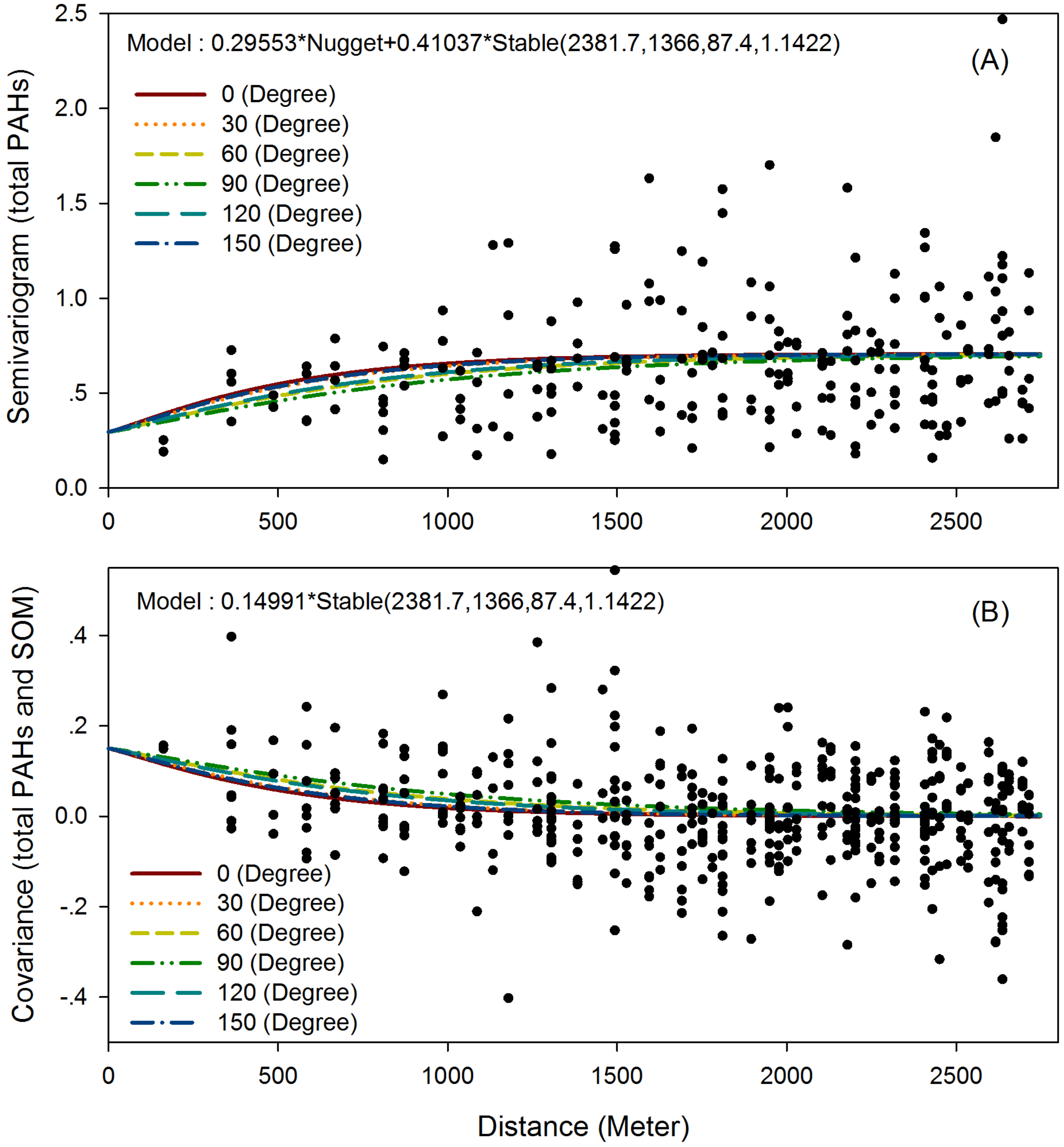

Fig.S3 Semivariogram and covariance plots with different search directions in the Cokriging interpolation analysis
